# Supplementary material for: Multi-scale Interaction Mechanism for Edge-Localized-Mode Suppression in the Tokamak Edge
Source: Nat Commun. 2025 Nov 21;16:11526. doi: 10.1038/s41467-025-66313-7 (PMC12748781; doi:10.1038/s41467-025-66313-7)
Supplement: Supplementary file 1 — Supplementary Information [file 41467_2025_66313_MOESM1_ESM.pdf]

Supplementary Document for "Multi-scale Interaction Mechanism for Edge-Localized-Mode Suppression in the Tokamak Edge"

Zeyu Li[1,*], P. H. Diamond[2,*], Xi Chen[1], F. Khabanov[3], Xueqiao Xu[4], R. J. Hong[5], V. S. Chan[6], C. M. Muscatello[1], L. Zeng[5], G. Y. Yu[7], T. Rhodes[5], G. R. McKee[3], Zheng Yan[3] and M. E. Austin[8]

[1]General Atomics, PO Box 85608, San Diego, CA 92186-5608, USA
[2]University of California, San Diego, CA 92093, USA
[3]University of Wisconsin-Madison, Madison, WI 53706, USA
[4]Lawrence Livermore National Laboratory, Livermore, CA 94550, USA
[5]University of California, Los Angeles, CA 90095, USA
[6]Institute of Plasma Physics, Chinese Academy of Sciences, Hefei, Anhui 230031, China
[7]University of California, Davis, CA 95616, USA
[8]University of Texas at Austin, Austin, TX 78712, USA

*Corresponding Author: lizeyu@fusion.gat.com; pdiamond@ucsd.edu

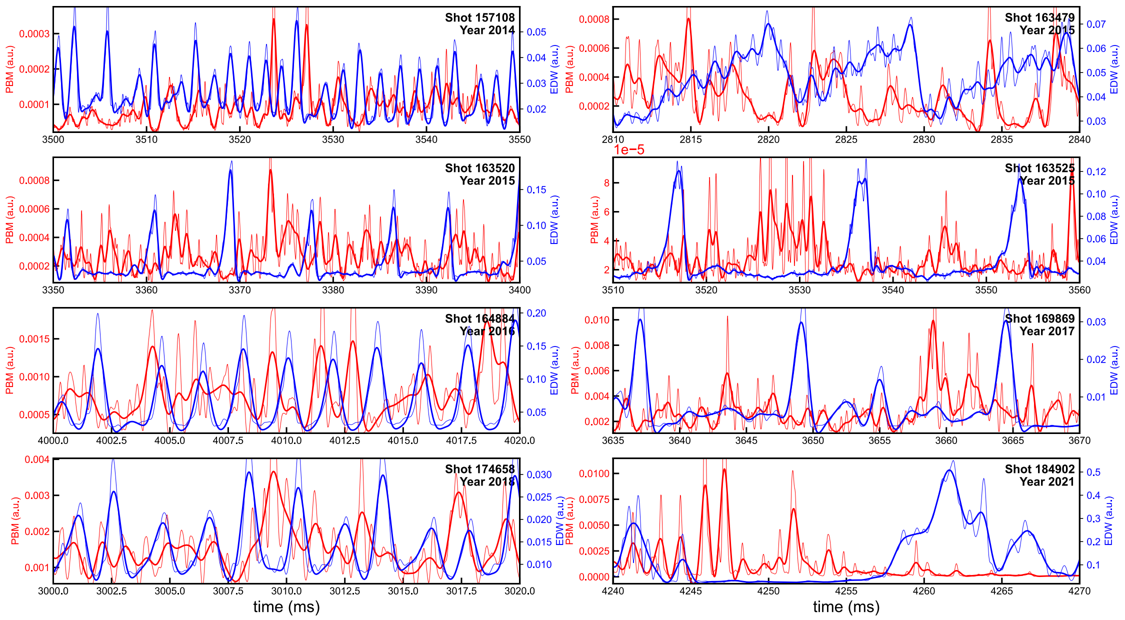

Supplementary Fig. 1. Additional examples of turbulence–MHD interactions observed in wide pedestal QH-mode experiments over the past few years. As shown in Fig. 1, the red curves represent the low-frequency (10–50 kHz) MHD mode measured by BES, while the blue curves correspond to the high-frequency (0.5–2 MHz) turbulence measured by DBS. The thin curves show the raw signals from the diagnostics, while the thick curves indicate the corresponding low-pass filtered data to highlight envelope modulation. Despite limitations in diagnostic resolution and interference from other perturbations, a consistent trend of turbulence–MHD interaction is observed across these discharges, underscoring the universality of this phenomenon.

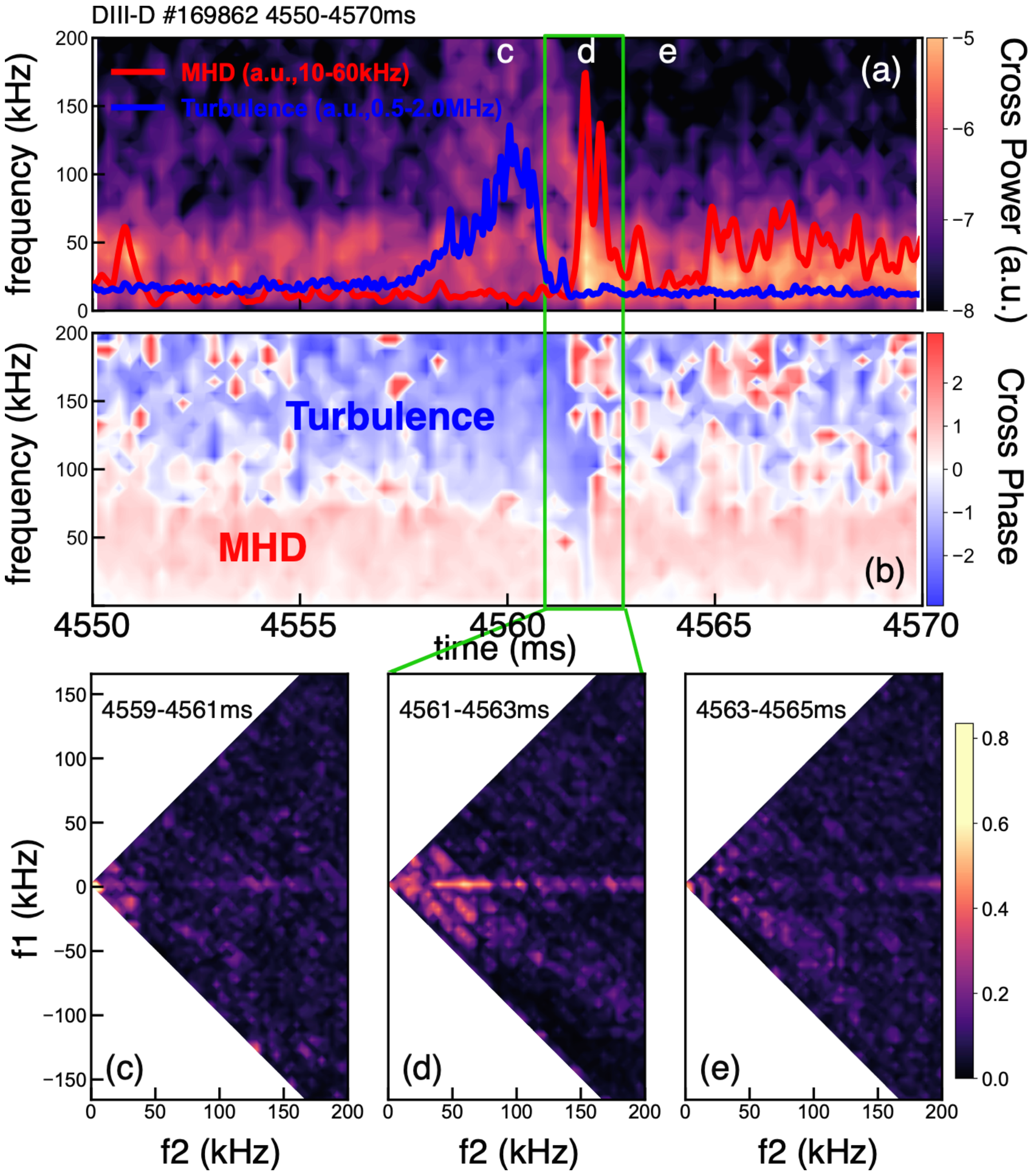

Supplementary Fig. 2. Additional cross-phase and bicoherence of the zoomed-in plot of one strong electron turbulence burst. (a) and (b) present the turbulence measurements in terms of cross-power and cross-phase, respectively, obtained from two poloidally adjacent channels at ψ_N~0.94 of the BES diagnostic. The positive cross phase represents the mode rotates in the ion diamagnetic drift direction (IDD) and the negative cross phase represents the electron diamagnetic drift direction (EDD). The bicoherence b^2 computed for (c) before; (d) during and (e) after the turbulence chirps down to the low-frequency branch.

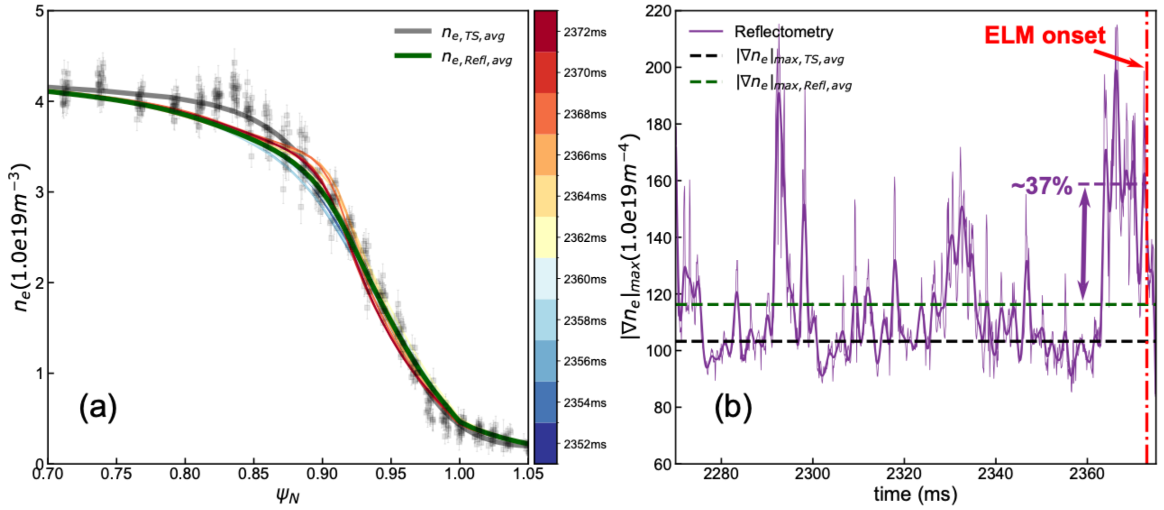

Supplementary Fig. 3. Evolution of the electron density profile and peak density gradient in wide-pedestal QH-mode discharge #173707. (a) Electron density profiles during the stationary phase (2270–2370 ms) of shot #173707. Black dots represent measurements from Thomson Scattering (TS), identical to those shown in Fig. 1 and Fig. 2. Error bars indicate instrumental uncertainty. The black curve is a fit to the TS data, while the dark green curve shows the time-averaged density profile from reflectometry over the same interval. Thinner colored curves (from blue to red) show the evolution of Reflectometry density profiles from 2352 ms to 2373 ms, each averaged over a ±1 ms window. (b) Temporal evolution of the peak electron density gradient from reflectometry. The black and dark green dashed lines represent the stationary-phase averages from TS and reflectometry, respectively. The red dash-dot line indicates the time of ELM onset (2373 ms). The peak density gradient increases by approximately 37% relative to the stationary phase in the final 10 ms preceding the ELM (2363–2373 ms).

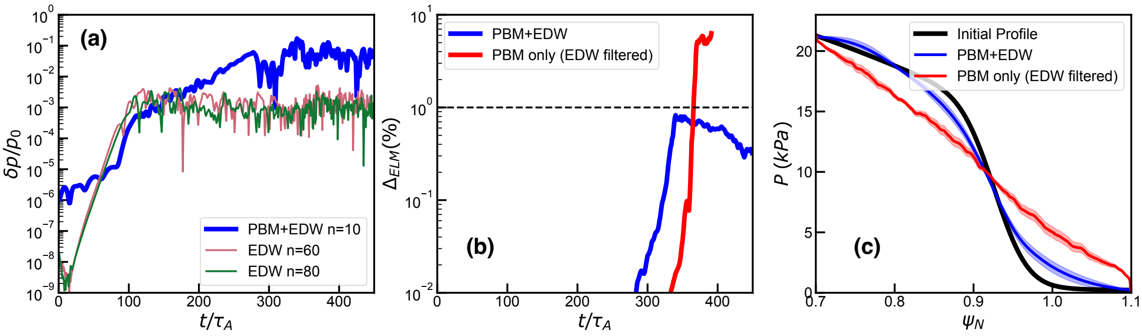

Supplementary Fig. 4. Additional BOUT++ simulation results showing high-n mode evolution, ELM size, and saturated pedestal profiles. (a) Nonlinear evolution of the low-n (n = 10) and high-n modes (n = 60 and 80). The high-n modes grow first and saturate at relatively low amplitudes, contributing little to transport. However, their early growth interferes with the development of the low-n mode, ultimately limiting the n = 10 PBM saturation level to $\delta p/p_0 \sim 0.06$; (b) Temporal evolution of the ELM size shown on a logarithmic scale, defined as $\Delta_{ELM} = \int_{\psi_N=0.7}^{Peak\ Gradient} (p_0 - \langle p \rangle)\, dV / \int_{\psi_N=0.7}^{\psi_N=1.1} p_0 dV$, where p_0 is the initial pressure profile and $\langle p \rangle$ is the nonlinearly evolved pressure. Red and blue curves correspond to simulations with PBM+EDW and PBM-only cases, respectively. The ELM size remains below 1% in the PBM+EDW case, while it reaches ~5% in the PBM-only case. (c) Time-averaged nonlinear saturated pedestal pressure profiles. The averaging interval corresponds to the saturation phase shown in Fig. 5. The PBM+EDW case preserves the pedestal shape, whereas the PBM-only case exhibits a pronounced collapse.

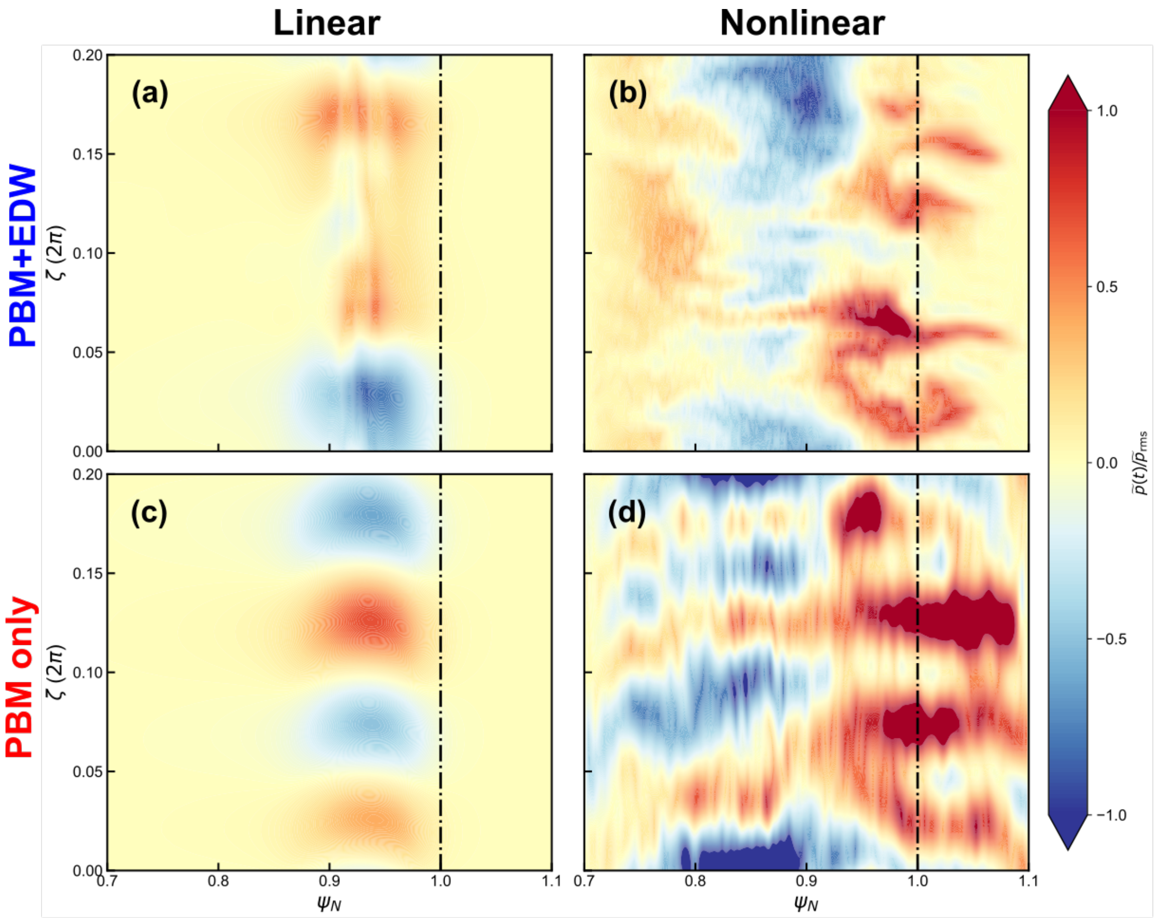

Supplementary Fig. 5. Additional BOUT++ simulations of mode eddies in the radial (ψ_N) and toroidal (ζ) directions. (a, b) Snapshots at the linear (t=200τ_A) and nonlinear (t=400τ_A) phases of the PBM+EDW case. (c, d) Corresponding linear (t=200τ_A) and nonlinear (t=390τ_A) phases of the PBM-only case. The simulations span 1/5 of the toroidal domain. In the PBM-only case (c), the dominant linear mode is n = 10, forming regular eddies. In contrast, the presence of turbulence in the PBM+EDW case (a) distorts the linear eddies through turbulence–MHD interaction. Nonlinear evolution reveals a marked difference: in the PBM+EDW case, the pedestal remains in a turbulence-mixed state, while the PBM-only case exhibits strong outward-propagating eddies and pedestal collapse, indicative of enhanced transport.

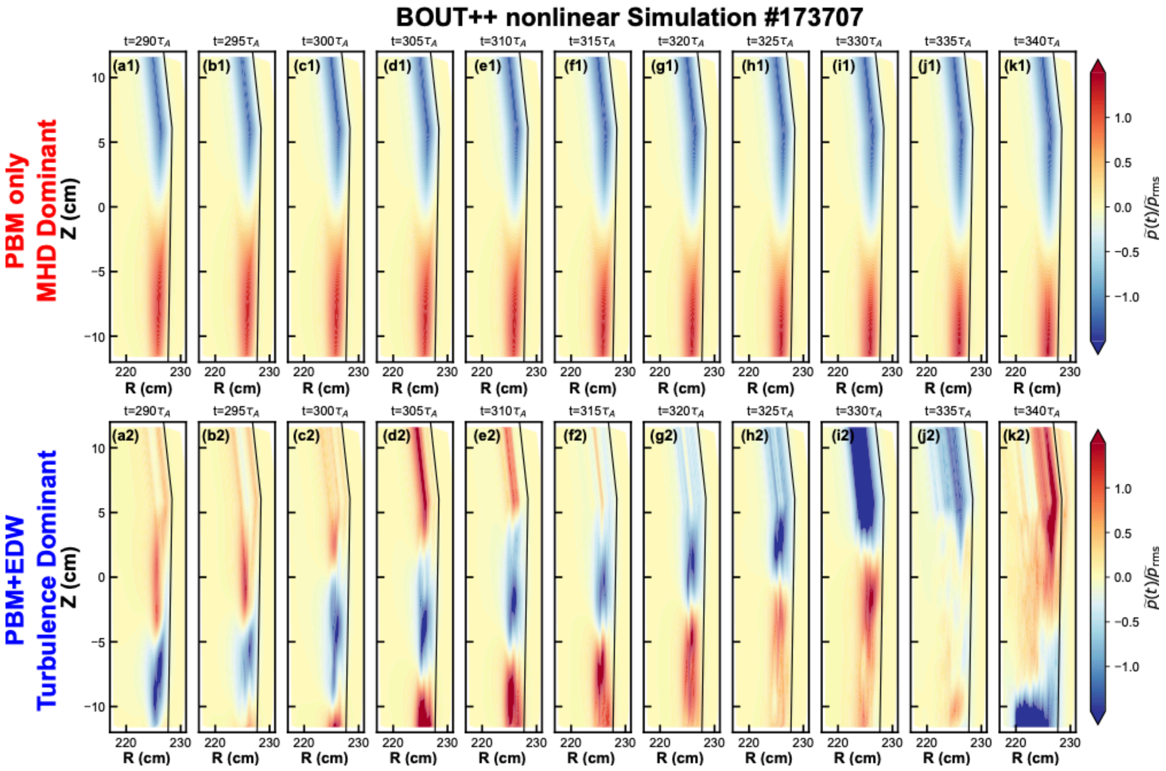

Supplementary Fig. 6. BOUT++ simulation of mode eddy evolution in a 2D poloidal (R–Z) slice. Top-row panels (a1–k1) show successive $5\tau_A$ (0.87μs) snapshots of the normalized pressure fluctuation $\tilde{p}/\tilde{p}_{rms}$ in the R–Z plane from t=$290\tau_A$ to t=$340\tau_A$ for the PBM-only (MHD-dominant) case. Bottom-row panels (a2–k2) present the same sequence for the PBM+EDW (turbulence-dominant) case. As in Fig. 3, the blue-to-red shading denotes negative-to-positive perturbations, the black curve marks the normalized separatrix (ψ_N=1). These simulations are based on the wide-pedestal QH-mode discharge #173707, which differs from the experimental case in Fig. 3. Nevertheless, similar features are observed, including mode rotation reversal and eddy shrinkage associated with turbulence–MHD interaction.
